# Mismatch between Conventional Femoral Arthroplasty Stems and Hip Morphology in the Elderly Chinese Hip Fracture Population

**DOI:** 10.5704/MOJ.2107.015

**Published:** 2021-07

**Authors:** JWX Siow, EBK Kwek

**Affiliations:** 1 Department of Orthopaedic Surgery, Tan Tock Seng Hospital, Singapore; 2 Department of Orthopaedic Surgery, Woodlands Health Campus, Singapore

**Keywords:** femoral stems, hip arthroplasty, hip morphology, medial femoral offset, mismatch

## Abstract

**Introduction:**

The morphology of the proximal femur differs in various populations. Based on our clinical experience, conventional femoral stems used in hip arthroplasty do not fit the Chinese population well. Hence, this study aims to evaluate the suitability of conventional femoral stems in the elderly Chinese hip fracture population requiring hip arthroplasty and to establish if gender and age related differences exist within this population.

**Material and Methods:**

We retrospectively analysed radiographic data of 300 patients from a tertiary hospital’s geriatric hip fracture database who underwent either hip hemi-arthroplasties or total hip arthroplasties. Proximal femur morphological measurements were recorded, analysed and compared to that of commonly used femoral stems. Subgroup analysis was performed to compare age and gender related differences.

**Results:**

A total of 18.3% of the study population had a medial femoral offset (MFO) of less than 30mm, which is the smallest available offset for the implants studied. 22.6% of female and 3% of male subjects had MFOs that were less than 30mm. In our subgroup analysis, males had significantly larger femoral head diameters, MFO and vertical femoral offsets compared to females. Older subjects (75-90 years old) had significantly smaller femoral head diameters, vertical femoral offsets and neck shaft angles compared to younger subjects (60-75 years old).

**Conclusion:**

Commonly used femoral stem implants have measurements that do not suit our Chinese population with small medial femoral offsets. In addition, elderly males have significantly larger femoral head diameters, medial and vertical femoral offsets whereas older subjects have significantly smaller femoral head diameters, vertical femoral offsets and neck shaft angles.

## Introduction

The anatomy of the proximal femur is known to differ between different populations. There are several studies in the literature describing the proximal femur anatomy^1,2,3^.

However, there are currently no studies that report about implant suitability as well as gender and age related morphological differences in the Chinese population. Our anecdotal clinical experience suggests that commonly used femoral stem implants for hip arthroplasty in our hospital may be unsuitable for our local patients, in particular patients with small medial femoral offsets (MFO). In addition, we hypothesised that males would have larger proximal femurs, however, age would not have a significant impact on the morphological measurements.

Hence, the aim of our study is to find out if commonly used femoral stem implants suit the elderly Chinese hip fracture population, to quantify the proximal femur morphology and to evaluate differences between gender and age groups in this population. This will help us to understand if age would have a significant impact in morphology and may help us to better plan for arthroplasty surgery.

## Materials and Methods

We retrospectively reviewed consecutive patients from a hip fracture database from a single tertiary hospital based on STROBE guidelines. Ethics board approval was obtained prior to the start of this study. Subjects aged 60 to 90 years old, who were admitted from 2011 to 2015 for traumatic femoral neck fractures and underwent either a hip hemiarthroplasty or total hip arthroplasty were included. Sample size was based on a study with comparable population by Tang *et al*^3^. Chinese subjects who had previous contralateral hip arthroplasty, pathological fractures of the proximal femur, had gross deformity of the proximal femur and had malrotated femurs on radiographs were excluded from the study.

**Fig. 1:**
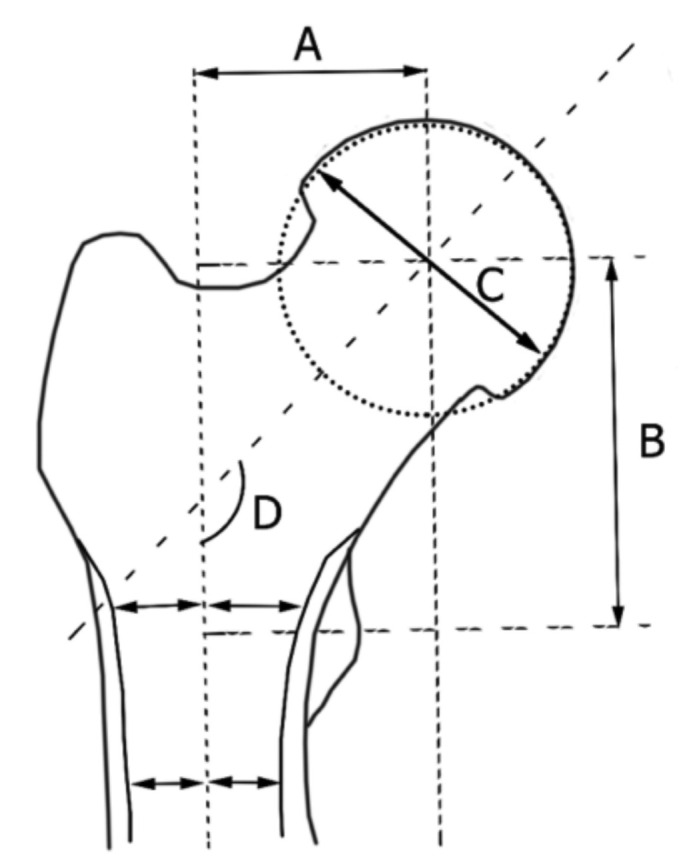
Illustration of proximal femur with morphological measurements. (A) medial femoral offset, (B) vertical femoral offset, (C) femoral head diameter, (D) neck shaft angle.

**Fig. 2:**
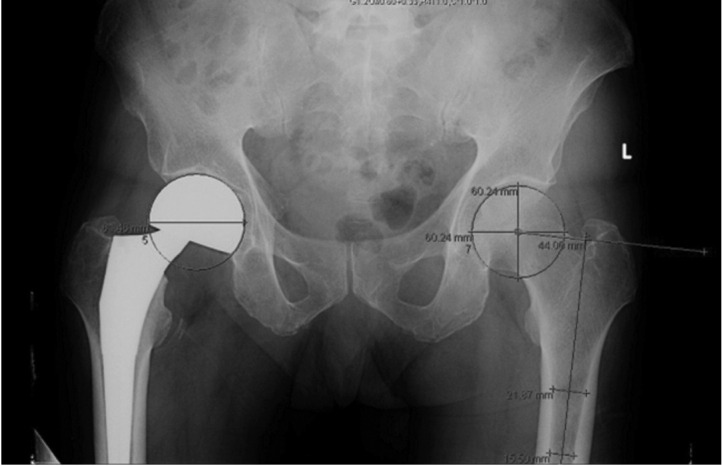
Measurement of medial femoral offset. Step 1: Calculate magnification error (measured shell diameter / actual shell diameter). Step 2: Identify hip centre. Step 3: Measure femoral head diameter. Step 4: Obtain anatomical axis of femoral diaphysis. Step 5: Using angular measurement tool to obtain perpendicular distance from femoral head centre and deriving the medial femoral offset.

**Fig. 3:**
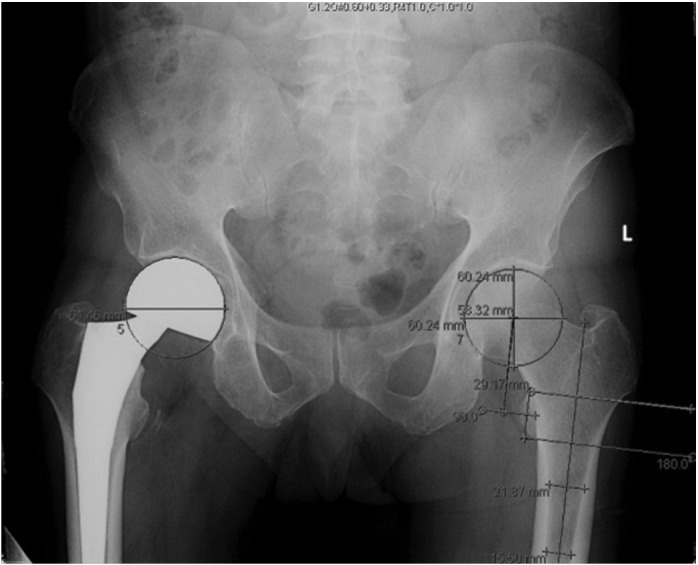
Measurement of vertical femoral offset. Step 6: Using the measurement tools to obtain the centre of lesser trochanter and measuring the vertical femoral offset.

The antero-posterior (AP) pelvic radiographs of the patients were measured (Fig. 1) using Centricity Enterprise Web Client V3.0 © by General Electric Healthcare and its measurement tools. We measured the MFO, femoral head diameter, vertical femoral offset as well as neck shaft angle of the proximal femurs. Magnification error was obtained by using known hemi-arthroplasty shell or total hip arthroplasty head sizes based on intra-operative records and taking those values divided by their digitally measured values. Measurements of the contralateral non-fractured hip was recorded (Fig. 2 and 3) and corrected accordingly based on the calculated magnification error. Subjects with malrotated femurs were excluded to allow for a more accurate assessment of the MFO and neck shaft angle. The commonly used femoral stem implants in our tertiary hospital are: Zimmer® M/L Taper [Zimmer, Inc. 1800 West Center Street Warsaw, Indiana 46580 USA], Zimmer ® Versys ® Fiber Metal Taper [Zimmer, Inc.,Warsaw, Indiana USA] and Zimmer® Advocate™ / Heritage™ cemented [Zimmer, Inc.,Warsaw, Indiana USA], Depuy Summit ® [DePuy Orthopaedics Inc., Warsaw Indiana, USA], Depuy Corail ® [DePuy Orthopaedics Inc., Warsaw Indiana, USA], Stryker® Accolade® [Stryker Corp, New Jersey, USA] and Stryker® Exeter™ [Stryker Corp, New Jersey, USA] hip prostheses. MFO ranges were obtained using implant guides and graphs depicting the range of MFO of subjects and implants were drawn using Microsoft ® Excel. In addition, to evaluate the differences between gender and age groups, we compared these variables: MFO, femoral head diameter, vertical femoral offset and neck shaft angle.

For statistical analyses, a two-tailed Student’s T test was conducted using STAT 13.0 [College Station, TX] and a p-value of less than 0.05 was considered significant.

## Results

**Fig. 4:**
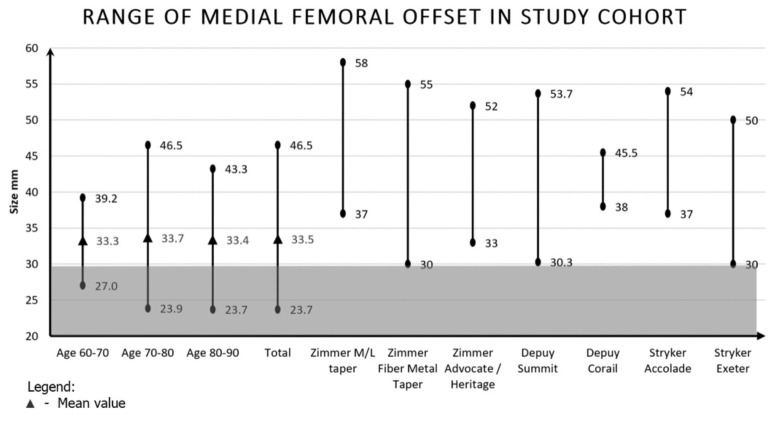
Range of medial femoral offset in study cohort. Legend: 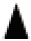
Mean value

A total of 1349 patients admitted for hip fractures between January 2011 and June 2015 were reviewed, of which 300 patients were included in our study. Of those included, 235 were females and 65 were males. A total of 1049 patients were excluded due to ethnicity, unsuitable radiographs and previous contralateral hip arthroplasties. The overall mean of the proximal femoral measurements were: femoral head diameter 43.5mm, MFO 33.5mm, vertical offset 48.7mm and neck shaft angle 130.8° (Table I). Comparison of the MFO of commonly used femoral stems implants with our study population showed that subjects with smaller MFO, notably those below 30mm, do not have implants with similarly small MFO for use (Fig. 4). With regards to suitability, the Stryker Exeter and Zimmer Fibre Metal Taper stems have MFO measurements that most closely match that of the study population.

**Table I:** Morphological measurements – comparing gender

Proximal femur measurements	Study Population (NT = 300)	Females (Nf = 235)	Gender Males (Nm = 65)	p-value
Femoral head diameter (mm)	43.5 (3.29)	42.5 (2.39)	47.3 (3.33)	<0.0001
Medial femoral offset (mm)	33.5 (3.68)	32.8 (3.27)	36.1 (3.94)	<0.0001
Vertical femoral offset (mm)	48.7 (5.16)	47.5 (4.40)	53.0 (5.4)	<0.0001
Neck shaft angle (degrees)	130.8 (3.96)	130.9 (4.17)	130.3 (3.05)	0.2795

Legend: Values represent mean values accompanied by standard deviations (in brackets) NT – Total population Nf – Total number of female subjects Nm – Total number of male subjects

In terms of gender differences, 22.6% of female subjects had a MFO of less than 30mm whereas only 3% of males had a MFO lower than 30mm. The mean offset value for male patients was smaller than the smallest available offset for three implants [Zimmer M/L taper, Depuy Corail and Stryker Accolade hip stem prostheses] and the mean value for female patients was smaller than the smallest available offset for four implants [Zimmer M/L taper, Zimmer Advocate/Heritage, Depuy Corail and Stryker Accolade hip stem prostheses]. This was attributed to male subjects having significantly larger femur sizes - they had significantly larger head diameters, MFO and vertical femoral offsets (Table I). In addition, older (75 - 90 years old) subjects had significantly smaller femoral head diameters, vertical femoral offsets and neck shaft angles (Table II).

**Table II:** Morphological measurements – comparing age groups

Proximal femur measurements	Study Population (NT = 300)	60 - 75	Gender 75 - 90	p-value
Femoral head diameter (mm)	43.5 (3.29)	42.5 (2.39)	47.3 (3.33)	<0.0001
Medial femoral offset (mm)	33.5 (3.68)	32.8 (3.27)	36.1 (3.94)	<0.0001
Vertical femoral offset (mm)	48.7 (5.16)	47.5 (4.40)	53.0 (5.4)	<0.0001
Neck shaft angle (degrees)	130.8 (3.96)	130.9 (4.17)	130.3 (3.05)	0.2795

Legend: Values represent mean values accompanied by standard deviations (in brackets) NT – Total population Nf – Total number of female subjects Nm – Total number of male subjects

## Discussion

The morphology of the proximal femur is known to differ between and within populations. Umer *et al*^1^ studied the differences between the Pakistani population and the western population and found significant differences in neck shaft angles and canal flare indices. To date, there has been only one retrospective study^3^ that addresses the morphology of proximal femur in Asians. In addition, there have been no studies comparing proximal femur morphology of various populations to conventional femoral stems for hip arthroplasty. Knowing the proximal femur morphology is important, as it will aid in pre-operative planning. In addition, knowing the limitations of the commonly used implants is also important as it will further help the surgeon to prepare and anticipate intra-operative challenges. Relevantly, our results show that there is a significant proportion of patients in our Chinese population that do not have a suitable implant sizes to match their low MFO.

Medial femoral offset (MFO) is defined as the shortest distance between the femoral rotation centre and the longitudinal axis of the proximal femur^4^ and is the distance of the biomechanical lever arm for the hip abductor muscles^5^. Much is known in the literature about the importance of restoration of the MFO especially for total hip arthroplasty^6^ but that for hip hemi-arthroplasty is still scant. In a recent prospective observational study^7^, restoration of medial femoral offset was also shown to correlate with the functional outcomes after hip hemi-arthroplasty. It is important because it affects the tension and moment arm of the abductor muscles, tension of the soft tissues^8^ and the amount of wear of the acetabular component^4,9^ – due to alterations in joint reaction forces. Biomechanically, an inadequate restoration of MFO will result in instability, increase in joint reaction forces, which will in turn increase polyethylene wear and cause loosening. On the contrary, an increased MFO will improve stability, increase moment arm of abductor muscles, reduce joint reaction forces and in turn decrease polyethylene wear^8,9^. One potential downside to increasing the MFO is higher rates of lateral hip pain^10^ though current evidence is conflicting, with some studies showing no correlation^11,12^. Therefore, further prospective studies will be required to establish the relationship between MFO and lateral hip pain.

In subjects with MFO that are smaller than the smallest available implant size with the smallest offset (30mm), intra-operative adjustments to modular components namely the vertical offset at the neck can be made to ensure proper tensioning of the abductor muscles. The other methods to adjust the vertical offset would be to alter the femoral neck cut for press fit stems and adjustment of stem insertion depth in cemented stems. These should ideally be templated pre-operatively. In terms of adjustment of MFO per se, all three commonly used implants have standard and extended offset options but do not have the option of a reduced offset. For this subgroup of patients with an offset of less than 30mm, vertical offset may have to be reduced to maintain abductor tension which may result in limb length discrepancy. Therefore, we recommend a reduced offset implant for the Chinese population, especially in the wake of increasing incidence of osteoporosis and hip fractures in Asia.

Limitations of our study are that digital radiographs used are two-dimensional and did not include radiographic markers. Therefore, only patients with a hip hemi-arthroplasty or total hip arthroplasty were selected as the implants have a spherical design of known dimension which acts as a “calibration marker”. In addition, rotational alignment of the femoral neck cannot be accurately assessed with radiography but can be more accurately and reliably measured with computerised tomographic (CT) scan^13,14^. Moreover, due to our study being retrospective, we were not able to obtain data on functional outcomes after hip arthroplasties. Finally, as our study population is Chinese, the results cannot be generalised to other ethnic groups.

Strengths of this study are that it is the first to show that conventional Western implants may not be suitable for the Chinese population. There are notable differences between the MFO of femoral stem implants used and the native MFO of subjects. This paves the way for further prospective studies to evaluate such differences.

## Conclusion

Our study shows that commonly used femoral stem implants for hip are unsuitable for the elderly Chinese hip fracture population with a low medial femoral offsets of less than 30mm, in particular for female patients. In addition, females and older patients (age 75 to 90 years old) have significantly smaller femoral head diameters, vertical femoral offsets and neck shaft angles.
